# Effects of calcium chloride and formalin injection into mediastinum testis on testosterone level, testicular tissue and semen quality in dogs

**DOI:** 10.1590/1984-3143-AR2025-0041

**Published:** 2026-01-30

**Authors:** Zehra Coşkun Bahçe, Fikret Karaca

**Affiliations:** 1 Department of Reproduction and Artificial Insemination, Faculty of Veterinary Medicine, University of Bingöl, Bingöl, Türkiye; 2 Department of Reproduction and Artificial Insemination, Faculty of Veterinary Medicine, University of Hatay Mustafa Kemal, Hatay, Türkiye

**Keywords:** chemical castration, testicular function, ultrasound

## Abstract

Intratesticular injection of chemical agents in dogs has been investigated for nearly half a century as promising methods for nonsurgical sterilization. This study was conducted to determine the effects of formalin and calcium chloride injection in mediastinum testis on semen quality, testicular width, testosterone level and histopathological changes in dogs, and to evaluate the usability and success of the intra mediastinum testis injection technique in chemical castration. Eighteen adult male dogs were divided into 3 groups including a control (0.5 ml 0.9% saline solution), formalin (5%) and CaCl_2_ (50%) into the mediastinum. Testicular diameter, spermatological characteristics and testosterone concentrations were determined on days 0, 22 and 44 following applications. The dogs were castrated on 45 day and the testicles were sent to the laboratory for histopathological examinations. Formalin and CaCl_2_ administered groups showed apparent enlargement and firmness of the testis with symptoms of discomfort and pain. One dog in the formalin group and two dogs in the CaCl_2_ group developed scrotal inflammation within three days, which progressed to scrotal ulceration and fistula formation. On day 44 after treatment, it observed that sperm motility and sperm concentration in the formalin and CaCl_2_ groups were decreased compare to control group while dead and abnormal sperm rates were increased (*P*<0.05). The testosterone concentration significantly increased in CaCl_2_ compare to control and formalin groups on day 44 (*P*<0.05). In histopathological evaluations, moderate degenerative changes were detected in the testicular tissue of the formalin group. The findings of this study showed that CaCl_2_ and formalin injections into the mediastinum testis under ultrasonography guidance are easily to perform and could be alternative to both the intratesticular injection and the surgical technique.

## Introduction

Dogs are one of the most common animal species in the world. However, it is known that very few of them are regularly examined by a veterinarian. The uncontrolled growth of the dog population negatively impacts public health, socioeconomics, politics and animal welfare (Downes et al., 2009; Salamanca et al., 2011). To address the rapid increase in the stray dog population, ongoing global research aims to control the reproduction of both male and female dogs. For controlling reproduction in male dogs, effective, safe, economical, and easy-to-apply techniques are desired. Intratesticular injection of chemical agents in dogs has been investigated for nearly half a century as a promising method of nonsurgical sterilization (Koger, 1977; Samanta, 1998; Singh et al., 2020; Leoci et al., 2014). These chemical agents affect testicular tissue by the reducing testicular blood flow, leading to degeneration, atrophy and deterioration of testicular functions (Ijaz et al., 2000). Various chemicals such as formalin (Bakır et al., 2006), glycerol (Immegart and Threlfall, 2000), lactic acid (Fordyce et al., 1989), calcium chloride (Jana and Samanta, 2007), zinc gluconate (Rafatmah et al., 2019) and hypertonic NaCl solution (Canpolat et al., 2015) have been investigated for non-surgical sterilization in dogs. Among these, intratesticular calcium chloride injection has attracted the most attention from researchers. Additionally, Moustafa et al. (2023) reported that intratesticular injection of 10% formalin (2 ml) appears successful for chemical sterilization of dogs and is applicable on a broad scale. The aim of this study was to administer the sclerosing/necrotizing agent directly into the mediastinum testis, where the rete testis is located. By impairing the rete testis ducts and allowing the chemicals to spread along the sperm-producing ducts, sperm and seminiferous tubules become non-functional, rendering the dog sterile. To date, no research has been conducted on applying sclerosing/necrotizing agents specifically to the mediastinum testis region for sterilizing dogs. The presented study was conducted to examine the effect of ultrasound-guided injection of CaCl_2_ and formalin into the mediastinum testis region on semen quality, testosterone levels and testicular tissue.

## Methods

### Experimental animals

Dogs with normal scrotal structures, confirmed through physical and ultrasound examinations, were included in the study. Dogs in each experimental group were housed freely in semi-open paddocks of 18.5 square meters. They were fed twice a day, had access to water at all times, and were kept under routine clinical observation for 45 days.

### Physical and ultrasound examinations

Dogs were held in a dorsal recumbent position, and the scrotum was inspected for size, symmetry, skin integrity, hair cover and pigmentation. Testicular consistency, elasticity, pain sensitivity, temperature changes and mobility were evaluated by palpation. Testis width was measured using Podany's testimeter on days 0, 22 and 44 as an index of testicular size. Ultrasound examinations were performed with a 5.0 to 7.5 MHz linear transducer (Hasvet 838 Ultrasound Scanner, Turkey).

### Experimental protocol

Dogs (n=18) were divided into tree experimental groups (n= 6 per group): control, formalin and calcium chloride groups. The control group received 0.5 ml of saline solution, while the experimental groups received either formalin (5%) or CaCl_2_ (50%) injected into the mediastinum testis under ultrasound guidance.

### Intra mediastinum testis injection

**Figure 1:**
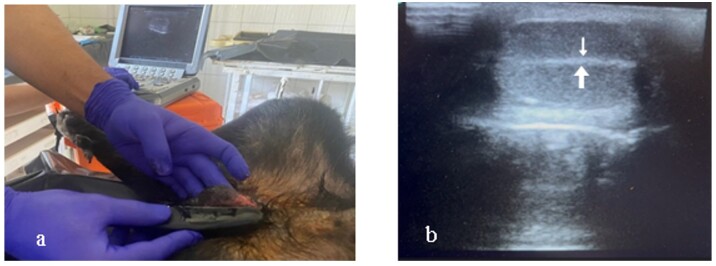
a) The position on the operating table of the dog and ultrasound examination in longitudinal plane of testis. b) The ultrasound image of the mediastinum testis (white arrows).

**Figure 2:**
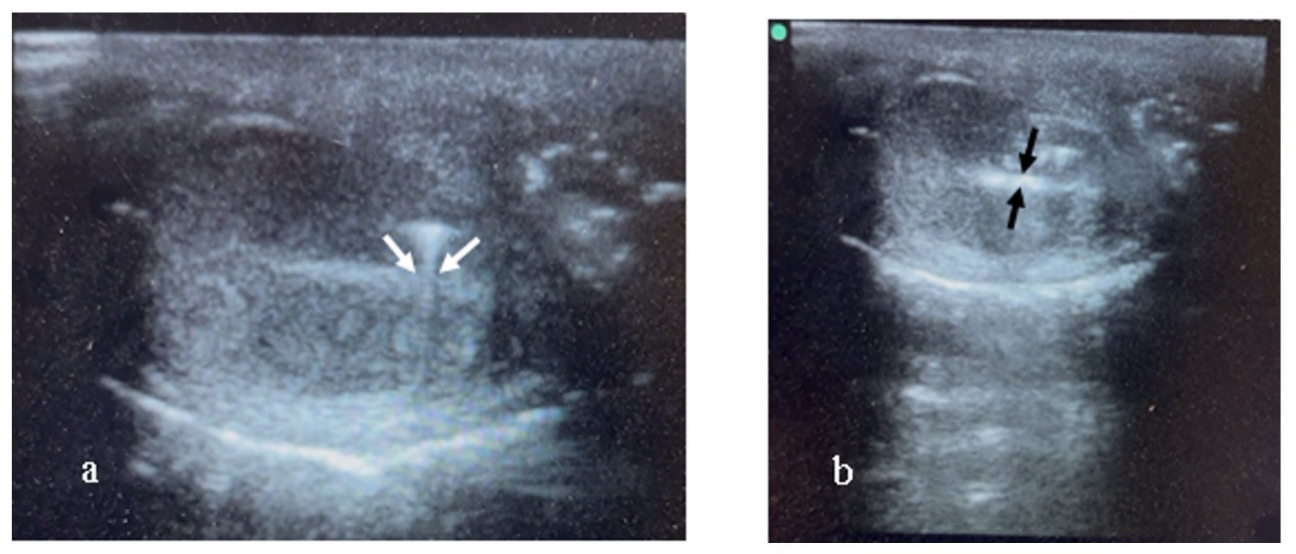
a) The insertion in the mediastinum testis with the needle of injection under the guidance of ultrasound image (white arrows). b) The ultrasound appearance of mediastinum testis (white arrows) shortly after injected in the mediastinum testis of the experimental solution (black arrows).

**Figure 3:**
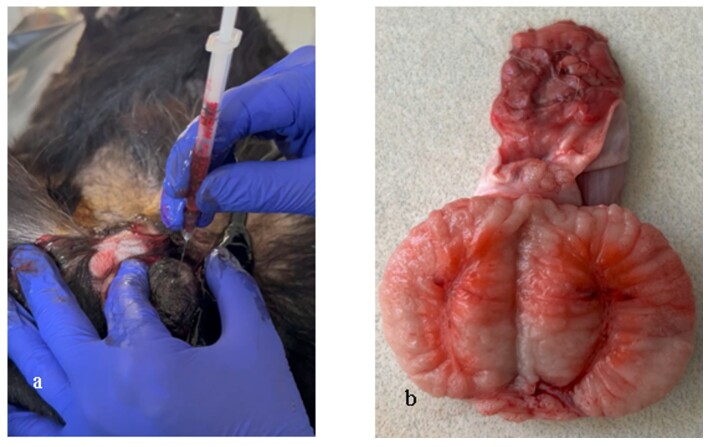
a) The holding together of the transducer and testis for ultrasound imaging, and injection region. b) The spreading throughout the mediastinum testis of solution after intra mediastinum testis injection of colorful solution (The macroscopic appearance with demonstration purpose).

General anesthesia was induced with intramuscular 2% xylazine HCl (Rompun, Bayer, Turkey) at 2-3 mg/kg and 10% ketamine (Ketasol, Interhas, Turkey) at 15-20 mg/kg. Anesthetized dogs were placed an operating table in a dorsal recumbent position. The scrotal hair was trimmed, and contact gel was applied. The ultrasound transducer was placed on the testis in longitudinal plane to visualize of the mediastinum testis (Figure 1a). The mediastinum testis was observed as hyperechoic line in central of the testis when examined in the longitudinal plane with 7.5 MHz linear transducer (Figure 1b). The ultrasound probe was held in place, and a sterile 24-gauge, 2.4 cm needle was inserted at the midpoint of the testis and advance under ultrasound guidance until reaching the mediastinum (Figure 2a). The chemical agents or saline solution were carefully injected into the mediastinum testis (Figure 2b). After the injection was completed, pressure was applied for 10 seconds to prevent leakage. To visualize the spread, 0.5 ml of saline colored with eosin solution was injected into one testis, and the dog was castrated within the following 30 minutes (Figure 3a). The testis was bisected longitudinally to observe to the chemical distribution (Figure 3b).

### Semen collection and evaluation

Dogs were accustomed to semen collection by digital massage, and at least two ejaculates were collected prior to the experiment. Semen was collected with the method reported by Freshman (2002). All three fractions of ejaculate were collected. Semen was evaluated for motility (%), sperm concentration (10^6^/ml), vital stain (% dead), and abnormal morphology rate (%).

The motility was evaluated as the percentage of sperm, moving in one direction (progressive motility) immediately after the collection of semen. For motility examination, a drop of semen was mixed with 3% sodium citrate and examined under 40x magnification of the light microscope. The average progressive motility in three fields was recorded (Taş, 2023).

Sperm concentration was measured using Makler counting chamber (SefiMedical, Israel) within 30 minutes after semen collection. For this, 10 μl semen sample was added to the Makler counting chamber at 37ºC. The count was done in an area of 10 squares from left to right, top to bottom, at 20x magnification. Sperm concentration was expressed sperm number in millions per milliliter.

Abnormal sperm rate was carried out staining with eosin-nigrosin of the sperm cells. A drop of the diluted sperm was mixed with 2 drops of eosin solution and 3 drops of nigrosin solution. Then, 1 drop of this mixture was taken and placed on the slide to prepare a smear. The slide was examined at 40x magnification under microscope. Approximately 200 sperm were examined in each smear sample, and abnormal morphology attached to the head, middle part and tail were recorded (Taş, 2023).

The eosin vital staining method was used to determine of the dead sperm rate. A small drop of semen sample was placed on the slide and was carefully mixed with an equal amount of eosin dye. A smear was drawn and allowed to dry in a short time. The preparation was examined under a light microscope at 40x magnification. A total of 400 sperm were counted under the microscope, starting from near the middle of the preparation. In the evaluation of these preparations; sperms whose heads had not received dye were considered alive, and those whose heads had received dye were considered dead (Taş, 2023).

### Testosterone concentration

Blood was collected from the cephalic vein on days 0, 22 and 44. After centrifugation at 3000 rpm for 5 minutes, serum was stored at -20°C. The serum testosterone level was measured using a rat testosterone (T) ELISA kit (Code: EA0023Ra), as recommended by the manufacturer (Bioassay Technology Laboratory) with a sensitivity of 0.043ng/ml and detection range of 0.1 – 40 ng/ml.

### Histopathology

At day 45, all dogs were surgically castrated for macroscopical and histopathological examination. Left and right testicular tissues were collected from each dog and fixed in 10% buffered formalin and Bouin's solution, and embedded in paraffine using the standard procedure. Paraffine blocks were sectioned at 4 μm and stained with hematoxylin and eosin. Sections were passed through graded alcohol (80%, 96% and 100%) and xylol again and examined under 100x and 400x magnifications using a light microscopy (Olympus CX31) and micro photographs (Olympus DP12) were taken.

### Data analysis

SPSS 23.0 package program was used in the statistical analysis of the study. One-Way Analysis of Variance (ANOVA) and Duncan’s multiple range test were used to assess group difference. For testosterone data, Shapiro-Wilk test was applied to evaluate the normality data using Phyton. ANOVA was performed to examine the differences between the groups and Post-Hoc Tukey HSD tests were applied for pairwise comparisons for groups with significant differences. A significance level of P < 0.05 was accepted.

### Ethics approval and consent to participate

Local Ethics Committee on Animal Experimentations of Hatay Mustafa Kemal University (No: 2020/04-35) has approved all the procedures in this study in accordance with the Türkiye by laws and World Organisation for Animal Health animal welfare standards for animal care, use in research and education.

## Results

The chemical agents or physiological saline solutions were successful injected into the mediastinum testis under ultrasound guidance. The injectability and spread of the solutions into the mediastinum testis are shown in Figure 2b. Macroscopic examination revealed that the mediastinum region was completely stained. In addition, the parenchymal tissue sorrounding the mediastinum testis was partially stained, with more intense staining in the injection region (Figure 2b).

Dogs in the formalin and CaCl_2_ groups showed apparent enlargement and firmness of the testis following intra mediastinum testis injections. Some of these dogs displayed signs of discomfort and licked the injection site. One dog (16,6%) in the formalin group and two dogs (33,3%) in the CaCl_2_ group developed scrotal inflammation within three days, which progressed to scrotal ulceration and fistula formation. These dogs were treated with anti-inflammatory drugs and antibiotics, and all wounds healed within two weeks. Dogs in the control group tolerated the intra mediastinum saline solution injection well.

On day 22, the mean testicular width increased in the formalin and CaCl_2_ groups compared to the control group. However, by day 44, the mean testicular width had decreased in the CaCl_2_ group compared to the formalin and control groups. The difference between the CaCl_2_ and formalin groups on day 44 was statistically significant (P˂0.05). As expected, testicular widths in the control group remained unchanged throughout the study (Table 1).

**Table 1:** Mean (± standard error) testicular widths obtained in control, formalin and CaCl_2_ group dogs on days 0, 22 and 44.

	**Testicular width (cm)**		
Groups	Day 0	Day 22	Day 44
Control (n=6)	5.5 ± 0.22	5.4 ± 0.3	5.5 ± 0.4ᵃᵇ
Formalin (n=6)	5.4 ± 0.1	6.3 ± 0.3	6.1 ± 0.2a
CaCl_2_ (n=6)	5.1 ± 0.2	5.5 ± 0.3	4.6 ± 0.4b

a, b Statistical difference is significant between groups marked with different letters in the same column (P < 0.05).

Before the intra mediastinum testis injections (day 0), no significant differences were detected among the control, formalin and CaCl_2_ groups in terms of sperm evaluation parameters. The sperm evaluation parameters recorded on days 0, 22 and 44 for each group are presented in Table 2. On day 22 following the intra mediastinum testis injections, the rates of dead and abnormal sperm were significantly higher in the formalin and CaCl_2_ groups compared to the control group (P < 0.05). The highest rate of dead sperm (54%) on the day 22 was observed in the CaCl_2_ group (P < 0.05).

**Table 2:** The mean (± standard error) sperm evaluation parameters of the control, formalin and CaCl_2_ group dogs on days 0, 22 and 44.

**Spermatological Parameters**	**Days**	**Control (n=6)**	**Formalin (n=6)**	**CaCl_2_ (n=6)**
**Sperm motility (%)**	Day 0	68.8±10.9	68.3±14.8	66.7±6.0
	Day 22	61.3±8.3	35.0±16.1	40.0±0.0
	Day 44	62.0±6.4ᵃ	10.0±7.6ᵇ	No Semen
**Sperm concentration (x10^6^/ml)**	Day 0	142.0±14.2	101.0±34.5	102.7±22.2
	Day 22	97.5±10.3	62.0±14.1	69.0±31.0
	Day 44	93.6±15.7ᵃ	24.3±14.9ᵇ	No Semen
**Abnormal sperm ratio (%)**	Day 0	4.5±1.6	6.3±1.5	5.0±1.2
	Day 22	9.0±0.9ᵃ	20.3±1.7ᵇ	34.7±8.3ᵇ
	Day 44	13.0±2.5ᵃ	24.3±2.7ᵇ	No Semen
**Dead sperm ratio (%)**	Day 0	13.5±2.7	14.0±3.8	14.7±2.0
	Day 22	14.8±1.8ᵃ	35.7±2.6ᵇ	54.0±11.0ᶜ
	Day 44	19.2±2.3ᵃ	37.7±2.6ᵇ	No Semen

a, b Statistical difference is significant between groups marked with different letters in the same rows (P < 0.05).

On day 44 after intratesticular injection, no semen could be collected from any dog in the CaCl_2_ group. Four of these dogs showed no penile erection during digital massage, while two had mild erections. Despite these findings, the dogs remained clinically healthy, and their testosterone concentrations were high. Significant differences in all sperm parameters were found on day 44 between the formalin-treated group and the control group (P < 0.05).

The serum testosterone concentrations on days 0, 22 and 44 in the study groups are shown in Table 3. On day 0, serum testosterone levels were similar in the control, formalin and CaCl_2_ groups. On day 22, mean testosterone concentrations in the control, formalin and CaCl_2_ groups were 10.6±0.6, 8.6±1.7 and 12.2±0.8 ng/ml, respectively, with significant difference observed between the formalin and CaCl_2_ groups (P < 0.05). On day 44, the mean testosterone concentration in the CaCl_2_ group was significantly higher than those in the control and formalin groups (P < 0.05).

**Table 3:** Mean (± standard error) testosterone concentrations obtained on days 0, 22 and 44 in the study groups.

	**Testosterone Concentration (ng/ml)**		
**Groups**	Day 0	Day 22	Day 44
**Control (n=6)**	9.3±1.6ᴬ	10.6±0.6ᵃᵇᴬ	7.5±1.6ᵃᴮ
**Formalin (n=6)**	9.4±1.9ᴬ	8.6±1.7^aB^	8.0±1.1ᵃᴮ
**CaCl_2_ (n=6)**	11.6±1.3ᴬ	12.2±0.8^bB^	11.1±0.6ᵇᴬ

a, b Statistical difference is significant between groups marked with different letters in the same column (P < 0.05); ^A, B^Statistical difference is significant between groups marked with different letters in the same rows (P < 0.05).

At 45 days post-injection, all dogs were surgically castrated for histopathological examination. Testes from the control group were apparently normal macroscopically. However, histopathological evaluation revealed that seminiferous tubules in these dogs were lined with a reduced number of spermatagonia and Sertoli cells, and the lumens were narrower and irregular than normal. On the other hands no marked reduction in testis size or fibrous tissue formation was observed in the control group dogs.

## Discussion

The goal of this study was to evaluate if intra mediastinum testis injection of CaCl_2_ or formalin solutions is an effective method for sterilizing free-roaming dogs. For this, it was investigated testicular size, sperm characteristics, serum testosterone concentration, and changes in testicular tissue. We evaluated feasibility and efficacy of injections into mediastinum testis of the chemical solutions. The chemical agents or saline solutions were successfully administered into the mediastinum testis under ultrasound guidance.

Previous studies reported testicular swelling 24 to 72 h following intratesticular injection of formalin (Bakır et al.*,* 2006; Moustafa et al., 2023) and CaCl_2_ (Jana and Samanta, 2007; Abu-Ahmed, 2015). Consistent with these findings, we also observed testicular enlargement within three days after the injections of the formalin and CaCl_2_ solutions. Silva et al. (2018) reported maximum swelling at 24 h after CaCl_2_ injection and reduced starting on day six. In our study, mean testicular widths increased in both the CaCl_2_ and formalin groups by day 22, but by day 44, a significant reduction was observed in the CaCl_2_ group compared to the formalin group (P < 0.05). In the dogs injected intra-testicular 1.0 ml of 20% CaCl_2_ solution, the mean values of all the parameters of testicular morphometry were found to be increased significantly from prior treatment to post-treatment day seven, followed by a significant decline by days 15 and 30 (Thakre et al., 2023). Moustafa et al. (2023) reported that the injections of intratesticular formalin caused a decrease (atrophy) of the testis sizes of dogs within 60 days. Similarly, the previous studies were reported that the testis became atrophied approximately in 2 (Abu-Ahmed, 2015) or 3 months (Leoci et al., 2019) after intratesticular injection of the CaCl_2_. Morphological and morphometric changes observed in testes of CaCl_2_ and formalin injected groups found in the present study was consistent with these findings.

Sperm evaluation parameters were adversely affected in both treatment groups. By day 22, we observed increased rates of dead and abnormal sperm, with reduced sperm motility and concentration. Moustafa et al. (2023) reported that the semen samples collected from the epididymis after 60 days from intratesticular formalin (10%) injections did not include sperm. On the other hand, Silva et al. (2018) reported that 6 dogs treated with 7.5% CaCl_2_ solution combined with 0.5% DMSO presented azoospermia on day 30. All dogs treated with intratesticular injection of 20% CaCl_2_ in 95% ethanol become sterile with azoospermia achieved over the 9-month study (Leoci et al., 2019). In our study, the formalin and CaCl_2_ groups were the oligospermic on day 22 following treatment. This difference could be related to the sperm collection method and time, application site and combination and dose of chemical agents (Leoci et al., 2019; Silva et al., 2018, Moustafa et al., 2023). However, the sperm motility (40%) and sperm concentration (69.0±31.0 x10^6^/ml) obtained on the day 22 in the CaCl_2_ group were consistent with the sperm motility (13.3%) and sperm concentration (108.0 ± 79.3 ×10^6^/ml) on the day 30 after the treatment reported by Silva et al. (2018). In the CaCl_2_ group, no semen could be collected on day 44. The reason why semen could not be collected from dogs can be speculated as the lack of libido or to the volume (0.5 ml) and concentration (50%) of the CaCl_2_ injected into the mediastinum testis. Leoci et al. (2019) reported that the sexual behaviors were not observed at month 9 after intratesticular injection of a solution of 20% CaCl_2_ in 95% ethanol in dogs. Furthermore, it was reported that intratesticular injections of 30% or 60% CaCl_2_ solutions resulted in azoospermia in all dogs over one year period (Leoci et al., 2014).

In the current study, the testosterone levels were above baseline level in all groups. the formalin and control groups had significant difference between testosterone levels on day 0 and 44 day. However, the testosterone levels of CaCl_2_ group were higher than those in the control and formalin groups on day 22 and 44. Our results were in disagreement with the reports of others (Moustafa et al., 2023; Jana and Samanta, 2007; Leoci et al., 2014; Abu-Ahmed, 2015; Leoci et al.*,* 2019). However, our results were in agreement with the reports of Nader et al. (2023) and Silva et al. (2018) who noted not significant effect on testosterone levels on days 21and 60 after intratesticular CaCl_2_ injection. Also, Vanderstichel et al. (2015) reported that in the chemically sterilized dogs found no significant difference of testosterone levels at the pre-treatment and the post-treatment. Similarly, testosterone levels have been reported to not affected in bulls (Pereira et al., 2018) and donkeys (Ibrahim et al., 2016) after following the intratesticular injection of CaCl_2_. In this study, the testosterone concentrations obtained the formalin and CaCl_2_ injected groups might be explained with a severe connective tissue increase in the intertubular areas or the unaffected of Leydig cells. İbrahim et al. (2016) reported that the absence of changes in serum testosterone levels after intratesticular CaCl_2_ injection in donkeys associated with a compensatory proliferation of Leydig cells in the histopathological sections. Further investigations are needed to clarify the effects of both intratesticular and intra mediastinal injections of chemicals sterilants on testosterone production.

The histopathological findings of the testicular tissues of CaCl_2_ and formalin groups were generally similar. However, the seminiferous tubules were lined with very few spermatagonia and Sertoli cells in the CaCl_2_ group, and their lumen was almost empty. Therefor it was determined that a severe connective tissue increase was formed in the intertubular area and replaced the testicular parenchymal tissue. The testis of the dogs in formalin group was not observed the coagulative necrosis of seminiferous tubules, dystrophic calcification and hemorrhages (Moustafa et al., 2023). The most of the histopathological findings observed in the CaCl_2_ group were similar to previous studies (Jana and Samanta, 2007; Chatterjee et al., 2009; Silva et al., 2018; Puri et al., 2018; Leoci et al., 2019; Nader et al., 2023; Thakre et al., 2023); except for calcified foci in the interstices (Silva et al., 2018), necrosis (Chatterjee et al., 2009; Puri et al., 2018) and degeneration (Thakre et al., 2023) of Leydig cells, and the coagulative necrosis in the seminiferous epithelium (Jana and Samanta, 2007; Puri et al., 2018). These differences may be related to the composition, dose (Silva et al., 2018), injection site of CaCl_2_ (Jana and Samanta, 2007; Chatterjee et al., 2009; Puri et al., 2018; Leoci et al., 2019; Thakre et al., 2023), and testicular histopathology time after treatment (Leoci et al., 2014; Leoci et al., 2019).

## Conclusion

The clinical observation, testicular size, sperm analysis and the histopathological examination findings revealed that the intra mediastinum testis injection technique could be alternative of both the intratesticular injection and the surgical technique. The injection of CaCl_2_ and formalin into mediastinum testis under ultrasonographic guidance could be easily performed, but there is a need for further studies with the efficacy of the injections in less amounts and concentrations of both chemicals in dogs. The results of this study also made us to think about the utilization of other chemical sterilization compounds in the same procedure for further investigations.

## Data Availability

Research data is available in the body of the article.
